# Real-Time Analysis of Changes in Internal and External Root Temperatures Using Different Systems for Activating the Irrigation Solution

**DOI:** 10.1155/ijod/3385512

**Published:** 2025-04-04

**Authors:** Maria Eduarda Paz Dotto, Julia Menezes Savaris, Luiz Carlos de Lima Dias-Junior, Tamer Ferreira Schmidt, Lucas da Fonseca Roberti Garcia, Cleonice da Silveira Teixeira, Eduardo Antunes Bortoluzzi

**Affiliations:** ^1^Department of Dentistry, Federal University of Santa Catarina, Florianópolis, Santa Catarina, Brazil; ^2^Department of Dentistry, University of Southern Santa Catarina, Palhoça, Santa Catarina, Brazil; ^3^Department of Diagnosis and Oral Health, University of Louisville, Louisville, Kentucky, USA

**Keywords:** endodontics, root canal irrigants, sodium hypochlorite, temperature, ultrasonic vibration

## Abstract

**Introduction:** There is a concern regarding the heating transfer to the periodontal tissues after irrigating solution activation. Therefore, this study analyzed the real-time changes in internal and external root temperatures using different systems for activating the irrigant.

**Methods:** Two single-rooted mandibular premolars were chemomechanically prepared. Three orifices were drilled on the root surface at 3, 6, and 9 mm from the apical foramen with a spherical diamond bur. In one tooth, drilling was restricted to the cementum. In another tooth, drilling was performed close to canal dentin. Thermocouple sensors were coupled to the orifices and fixed with resin for temperature measurement. Irrigation was performed with 2.5% NaOCl at 25°C or 45°C. The irrigant was activated for 20-, 30- and 60 s using 3 different systems: passive ultrasonic irrigation (PUI), Ultra X (UX), and endoactivator (EA). For each initial irrigant temperature, time, and activation system, the tests were repeated 8 times, resulting in a total of 96 evaluations for the external and internal root temperatures (*n* = 48 for each).

**Results:** Data was statistically analyzed with a multilevel linear regression model and intraclass correlation coefficients (ICCs) were calculated. Then, four-way ANOVA with Bonferroni's post hoc tests performed intergroup and intragroup comparisons. EA promoted lower temperature increase than PUI and UX (*p*  < 0.05). PUI and UX induced similar internal and external temperature changes when irrigated with NaOCl at 25°C..

**Conclusion:** The initial temperatures (25°C or 45°C) and the activation systems of the irrigant had influence on the internal and external radicular temperatures. The activation period had little influence on root temperature changes, which may be deemed clinically safe.

## 1. Introduction

The cleaning capacity of sodium hypochlorite (NaOCl) is potentiated by its concentration, usage period within the root canal, activation protocols, and temperature [1, 2 ]. In addition [3, 4 ], several studies have reported that increasing the temperature of NaOCl improves its reaction rate and flow, resulting in the faster dissolution of organic matter. The temperature of the irrigant can be raised by preheating or intracanal activation [2, 4 ]. Sonic and ultrasonic activation of the irrigant has been highly recommended to reduce the bacterial content during root canal shaping [1, 3 ]. Compared to conventional irrigation (CI) with a syringe and needle, activation of the irrigant leads to greater hydrodynamic flow in hard-to-reach areas of the root canal system [5, 6 ].

There is a genuine concern with the heating transfer to the periodontal tissues after irrigant preheating [7]. Temperatures above 47°C on the external root surface harm the periodontal ligament as they might decrease the blood flow and promote bone resorption [8]. Recently, a study [9] demonstrated that preheating the irrigant at 60°C during CI resulted in higher temperatures along the root canal than activation methods.

Some studies [2, 3, 9] have already verified the internal temperature of the irrigant during the solution activation process, but no one has investigated the external temperature of the root canal, which is an important factor to be observed due to the proximity to the periodontal tissue. Depending on the temperature reached, it can cause damage to adjacent tissue [7, 10]. Bearing this in mind, this study aimed to investigate in real time the temperature change along the root canal caused by the activation of the NaOCl at different periods (20, 30, and 60 s), at different initial temperatures of the irrigant solution (25°C and preheated at 45°C), and using different activation systems—passive ultrasonic irrigation (PUI), endoactivator (EA), and PUI with portable ultrasound Ultra X (UX). The null hypotheses tested were that there would be no significant difference among the different activation systems regarding (i) irrigant activation period, (ii) initial temperature of the irrigant, (iii) activation systems, and (iv) internal and external radicular temperatures.

## 2. Materials and Methods

### 2.1. Sample Size Calculation

**Figure 1:**
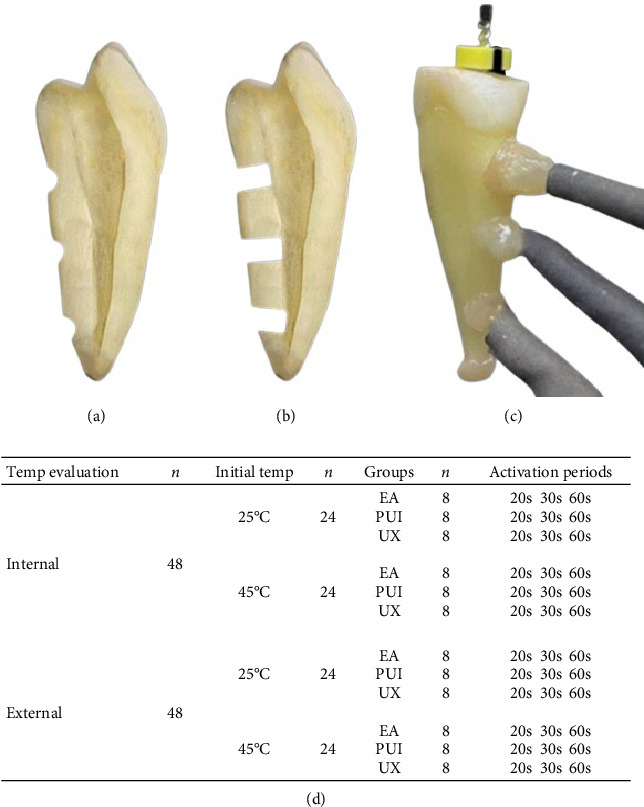
Representative images of the thermocouple adaptation at various root thirds and the methodological design of groups formation are shown. (a) Orifices drilled restricted to the cementum. (b) Orifices drilled until reaching dentin close to root canal. (c) Thermocouple sensors attached to the orifices with composite resin at different root canal thirds. Note the size 40 K-file positioned inside the root canal and the root apex covered with composite resin. (d) Methodological design of the experimental groups formed by different activation systems (EA, PUI, and UX) of the irrigant according to temperature evaluation (Internal or External), initial temperature of the irrigant (25°C and 45°C), and activation periods (20-, 30-, and 60 s). EA, endoactivator; PUI, passive ultrasonic irrigation; UX, Ultra X.

This study was previously approved by the Local Ethics Committee (No 4.942.278). Considering ANOVA and the F Family tests, the sample size was estimated based on a previous study [9] using G^*⁣*^*∗*^^Power 3.1.9.4 software (Heinrich-Heine Universität, Düsseldorf, Germany). The effect size was set at 0.58, with an alpha error of 0.05 and a beta power of 0.80. Considering two initial temperatures of the irrigant and three-irrigation activation systems, six groups were formed for each internal or external root temperatures evaluated (Figure 1). The total sample size for each internal or external root temperatures was 48, resulting in 8 repetitions per experimental group. Because it was a nondestructive method, allowing all comparisons to be made under the same structural conditions, two teeth were used for all analyses (one tooth for each root temperature evaluated, internal or external) [9].

### 2.2. Sample Selection

After periapical radiographs obtained in the mesiodistal and buccolingual directions, freshly extracted mandibular premolars, with a single and straight root canal (curvature < 5°), fully formed root, and without previous endodontic treatment, were selected. Furthermore, a stereoscopic magnifying glass with × 4 magnification was utilized for the evaluation of cracks or imperfections. Any teeth displaying indications of calcification, internal or external resorption, cracks, fractures, or carious lesions were replaced. Two teeth that met these selection criteria and exhibited similar rounded root canal morphology were chosen.

The root canals were accessed and then negotiated with a size 10 K-file (Dentsply Malillefer, Ballaigues, Switzerland). The tooth length was determined by the direct method until the tip of the file was visualized in the apical foramen. Next, the coronal portion of the teeth was flattened with a carborundum disk to standardize the length of the specimens. The working length (WL) was established at 1 mm short of the apical foramen. Root canal preparation was performed with file R40 (40/.06) (Reciproc, VDW, Munich, Germany), coupled to a 6 : 1 reducing contra-angle with electric motor (VDW Silver Reciproc) adjusted to the “RECIPROC ALL” function, according to the manufacturer's recommendation. The patency of the apical foramen was passively maintained with a size 10 K-file (Dentsply-Maillefer). Next, the teeth were irrigated with 2.5% NaOCl. At the end of the preparation, final irrigation with 3 mL of 17% EDTA (Merck, Darmstadt, Germany) was performed, followed by 3 mL of 2.5% NaOCl, both by CI with a 30 G needle and syringe (NaviTip, Ultradent, South Jordan, UT, USA). Then, the root canals were dried with absorbent paper points (Cell Pack, Dentsply). A single operator, a specialist in endodontics, performed all procedures.

### 2.3. Sample Preparation

Three orifices (1.5 mm in diameter) were drilled perpendicular to the long axis of the teeth at 3, 6, and 9 mm from the apical foramen with a spherical diamond bur (Komet, Lemgo, Germany). In one of the teeth, drilling was restricted to the cementum using half spherical drill (0.75 mm) (Figure 1a), and in another tooth, drilling was performed until reaching the root dentin (1.5 mm spherical drill) (Figure 1b). Thermocouple sensors (Type-K - GHM Messtechnik, Regenstauf, Germany) were attached to the orifices (Figure 1c) to allow the measurement of the external and internal radicular temperatures at the different root canal thirds (cervical, middle, and apical). The accuracy of the thermocouples was verified beforehand using an alcohol bulb thermometer within the 20°C–50°C range, before and after each experimental session, with temperatures recorded to the nearest degree Celsius [11].

The thermocouple sensors were attached to the orifices with composite resin (Empress Direct, Ivoclar, Schaan, Liechtenstein) to stabilize and create a closed space [11, 12]. A size 40 K-file (Dentsply Malillefer) was positioned inside the root canal to ensure no direct contact with the thermocouple sensor, which was checked radiographically. The root apex of the tooth was also covered with composite resin to avoid extravasation of the irrigant solution during the irrigation protocols (Figure 1c). Finally, the tooth was immersed up to the cementoenamel junction in thermostatic water bath (Ratek Instruments, Boronia, Australia) containing distilled water at a controlled temperature of 37°C throughout the experiment to simulate the body temperature [3, 9].

### 2.4. Initial Temperature of the Irrigant

Two previous irrigant solution temperatures (25°C and 45°C) were evaluated. Irrigation with the NaOCl at room temperature was performed using 2 mL of 2.5% NaOCl at 25°C delivered inside the root canal by CI, followed by simultaneous cannula aspiration during 1 min. Next, the root canal was filled with 0.5 mL of NaOCl at the same temperature for immediate activation. Irrigation with the preheated NaOCl was performed using 2 mL of 2.5% NaOCl preheated at 45°C delivered inside the root canal by CI, followed by simultaneous cannula aspiration for 1 min. The syringe was kept warm to maintain the irrigating solution temperature constant. Next, the root canal was filled with 0.5 mL of NaOCl at the same preheated temperature for immediate activation.

### 2.5. Irrigating Activation Protocols

The irrigant activation was performed in the evaluation of internal and external root temperatures using each previously prepared tooth, as shown in Figure 1 (a–d). Between each repetition, the teeth were maintained at 37°C for at least 10 min to standardize the initial temperature of the tooth structure. After filling the root canal with 0.5 mL of 2.5% NaOCl, with the initial temperature set at 25°C or 45°C, the activation protocols were performed as follows:

PUI group (*n* = 8): Activation with an ultrasonic insert (E1 Irrisonic; Helse Ultrasonics, Ocoee, FL), coupled to an ART-P6 (Piper) Compact Piezoelectric Scaler (Bonart Medical, New Taipei City, Taiwan) inserted 1 mm below the WL, in a back-and-forth motion, the amplitude of 2–3 mm, for 20, 30, and 60 s. The power of the ultrasonic device was adjusted following the manufacturer's recommendations.

EA group (*n* = 8): Activation with a specific polymer tip size 25.04, inserted 1 mm below the WL, coupled to the sonic activator (Dentsply Maillefer), at speed 3, in a back-and-forth motion, the amplitude of 2–3 mm, for 20-, 30-, and 60-s.

UX group (*n* = 8): Activation with a silver ultrasonic insert, attached to a wireless UX handpiece (Changzhou Sifary Medical Technology Co., Changzhou, China), adjusted to a high power of 45 kHz, inserted 1 mm below the WL, in a back-and-forth motion, the amplitude of 2–3 mm, for 20, 30, and 60 s.

### 2.6. Assessment of Temperature Changes

Temperature changes during the irrigation protocol were assessed in real-time with Type-K thermocouples coupled to a digital thermometer device (PerfectPrime, NY, USA). Recordings were made at each experimental step. For example, after delivering the irrigating solution at 25°C and 45°C, a reduction of 5°C was observed in the mean temperature of the specimen (37°C) before activation. In addition, the maximum values of internal and external root temperatures were recorded for each root third, period of activation, and initial temperature of the irrigant.

### 2.7. Statistical Analysis

The data were analyzed by the Statistical Package for the Social Sciences software (IBM SPSS 17.0 Statistics for Windows, Version 21.0. Armonk, NY, USA) and JAMOVI v. 2.2.5.0 (The Jamovi Project 2023, Sydney, Australia). For all analyses, a 5% significance level was adopted. Initially, the data were explored for normality using the Shapiro–Wilk test. Multilevel linear regression model was used to investigate the effects of the irrigation activation systems (EA, PUI, and UX groups) and initial temperature of the irrigant (25°C and 45°C) as explanatory factors associated with the internal and external root temperature. Also, the period of activation (20, 30, and 60 s) and root third (cervical, middle, and apical) were added to the model as cluster variables, and intraclass correlation coefficients (ICCs) were calculated. Then, four-way ANOVA with Bonferroni's post hoc tests were used to perform the intergroup and intragroup comparisons. Parameter estimates, along with 95% confidence intervals, were calculated. The results from internal and external root temperatures were separately analyzed.

## 3. Results

### 3.1. Internal Radicular Temperature

The linear regression analysis revealed that the activation systems (*F* = 799.8; *p*  < 0.001), the initial temperature of the irrigant (*F* = 316.7; *p*  < 0.001), and the interaction between these factors (*F* = 84.7; *p*  < 0.001) were significant predictors of the internal root temperature. The adjusted *R*^2^ value was 0.795. This indicates that 79.5% of the variance in the internal root temperature was explained by the activation systems and initial temperature of the irrigant. Regarding the cluster variables, the internal temperature was affected by both the root canal thirds (ICC = 0.0816; *p*  < 0.001) and the activation periods (ICC = 0.062; *p*  < 0.001), but the impact of these variables is very limited as very-low ICC values were found.

**Figure 2:**
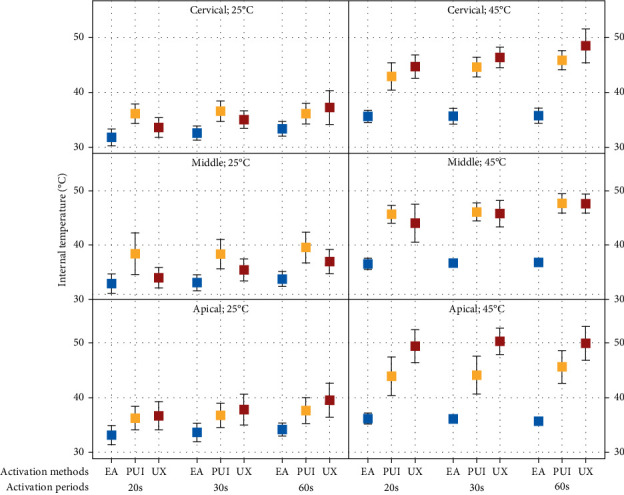
Internal analysis of initial temperature and root thirds between activation systems and periods pre-established.

In the four-way ANOVA analysis compares the activation systems (EA, PUI, and UX) with the initial temperature of the irrigant set at 25°C, statistical differences (*p*  < 0.05) were observed (Table 1). At the cervical and middle root thirds, there was a difference between EA and PUI within all activation periods (*p*  > 0.05) and between EA and UX only at the 60-s mark (*p*  < 0.05). In the apical third, PUI showed no statistically significant difference from the other activation systems (*p*  > 0.05), while UX differed from EA, demonstrating the highest temperatures across all activation periods (*p*  < 0.05) (Table 1, Figure 2).

When comparing the different activation methods with the initial temperature of the irrigant set at 45°C, EA exhibited the lowest temperatures (*p*  < 0.05) across all root thirds and activation periods (Table 1, Figure 2). There was no difference between PUI and UX at the cervical and middle thirds (*p*  > 0.05). However, at the apical third, all systems (EA, PUI and UX) showed differences among each other in all activation periods (20-, 30- and 60-s) (*p*  < 0.05).

Within each activation system and comparing the initial irrigant temperatures set at 25°C with those at 45°C (Table 1), EA demonstrated similarity between these initial temperatures at the cervical third only within 60 s of activation (*p*  > 0.05), and at the apical third, within 30- and 60-s intervals (*p*  > 0.05). Significant differences were observed between the initial temperatures for PUI and for UX, irrespective of the activation periods (*p*  < 0.05). Following the use of the activation systems, there was a tendency for an increase in tooth temperature from the cervical to the apical root canal thirds, mainly with the use of UX system at 45°C (Table 1, Figure 2).

### 3.2. External Radicular Temperature

The influence of the activation methods (*F* = 128.8; *p*  < 0.001), the initial temperature of the irrigant solution (*F* = 370.1; *p*  < 0.001), and the interaction between these factors (*F* = 22.8; *p*  < 0.001) as predictors of the external root temperature were shown by the linear regression analysis. The adjusted *R*^2^ value was 0.621, which indicates that 62.1% of the variance in the external root temperature was explained by the activation systems and initial temperature of the irrigant. The analysis of the cluster variables revealed that external temperature was not affected by the root thirds (*p*=0.101). On the contrary, the differences in activation periods (20, 30, and 60 s), significantly affected the external temperature (ICC = 0.0532; *p*  < 0.001), even though a very-low ICC value was shown.

In the four-way ANOVA analyses, when comparing the activation methods (EA, PUI, and UX) with the initial temperature of the irrigant set at 25°C (Table 2), significant differences were observed between EA and UX across all activation periods and root thirds (*p*  < 0.05). PUI and UX were statistically different from each other only at the middle root third, irrespective of the activation period (*p*  < 0.05). At the apical third, PUI differed from EA during the 30- and 60-s activation period (*p*  < 0.05).

When comparing the different activation methods with the initial temperature set at 45°C, EA and PUI showed no significant difference between each other, regardless of the activation period and root third (*p*  > 0.05). However, UX had statistical differences (*p*  < 0.05) at the middle third in all periods, when compared to EA and PUI. At the apical third, UX promoted the highest external radicular temperatures compared to EA in all periods, and when compared to PUI, UX differed from PUI at 30-s period (*p*  < 0.05).

**Figure 3:**
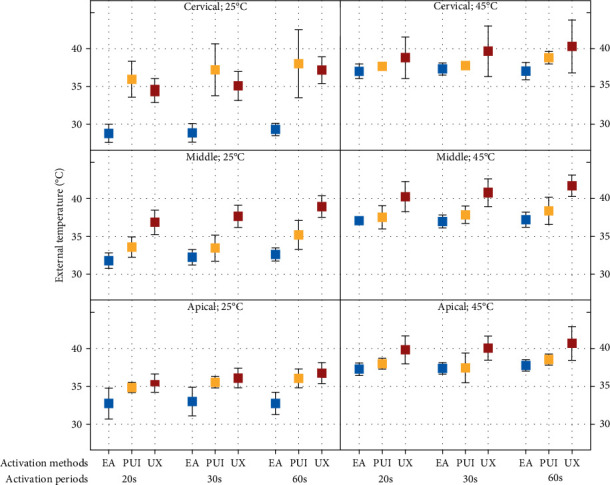
External analysis of initial temperature and root thirds between activation systems and periods pre-established.

Within each activation method and comparing the initial irrigant temperatures at 25°C with those at 45°C, PUI exhibited similarity between these initial temperatures at the cervical third, regardless of the activation period (*p*  > 0.05) (Table 2, Figure 3). At the middle third, all activation methods showed a significant difference (*p*  < 0.05) between 25°C and 45°C, irrespective of the activation period. Only when the activation period was 30 s at the apical third, PUI showed similarity between its initial temperatures (*p*  > 0.05) (Table 2) (Figure 3).

## 4. Discussion

This study aimed to assess temperature changes along root structures caused by the activation of irrigant at different periods (20, 30, and 60 s) initial temperatures of the irrigant (25°C and 45°C) and activation systems (EA, PUI, and UX). According to our findings, the null hypothesis was rejected, as the use of different irrigant activation systems significantly affected internal and external root temperatures, with these results also being influenced by the initial temperature and activation period of the irrigant. Additionally, the root third influenced the internal radicular temperature assessed. In light of these results, some considerations about the study should be discussed.

In the present study, the specimens were immersed in distilled water at 37°C to simulate the body temperature closely [3, 9, 13, 14]. Also, in order to achieve a highly controlled experimental setup, the temperature measurements were taken during the deposition and activation of the irrigant. This method enables the prediction of how the temperature changes with different initial temperatures of NaOCl [3]. The mean temperatures were recorded during irrigation and systems activation procedures. It was observed that there was a drop of ~5°C in the temperature of the specimens, when delivering the irrigant at 25°C and at 45°C. Other studies also demonstrated an intracanal temperature drop of 8°C [13] and 7.4°C [14], when root canal irrigation was performed with a syringe and needle, with the irrigant set at 25°C or at 20°C, respectively. In this study, to mitigate the temperature reduction, the syringe containing the preheated irrigant was kept in a water bath at 45°C until final irrigation occurred [9]. Even so, there was a drop in temperature. It can be assumed that the temperature of the external environment to the irrigant may have contributed to this drop, causing a faster heat dissipation [2, 13, 15].

The results of the present study demonstrated that, when the initial temperature of the irrigant was set at 45°C, EA exhibited the lowest mean internal temperatures compared to PUI and UX. This finding can be attributed to the fact that sonic devices operate at lower working frequencies than ultrasonic inserts [6]. The EA system operates at three different speeds, with the variance between the lowest operating frequency (160 Hz) and the highest (190 Hz) being minimal, resulting in minor alterations to the flow of irrigating solution within the root canal [6]. Conversely, activation with PUI and UX demonstrated pronounced intracanal heating in comparison to EA. Both PUI and UX utilize ultrasonic action to agitate the solution, generating micro-acoustic currents and cavitation phenomena [16, 17], thereby leading to localized temperature elevations [10]. UX, a wireless ultrasonic handpiece activator, provides versatility in frequency selection and compatibility with various inserts [16]. The metallic inserts employed in PUI and UX, characterized by smaller diameters and higher frequency oscillations, result in heightened temperatures compared to the more flexible and larger diameter polymer tip of EA [10, 16, 17].

The highest internal temperatures (over 50°C) were reached when UX was utilized at the apical third, across all activation periods, and when the irrigating solution was preheated to 45°C. These temperatures differed from those obtained with the other systems, PUI and EA. This can be attributed to UX's higher operating frequency (45 kHz) compared to PUI (26 32 kHz) and EA (160 190 Hz). Additionally, it can be assumed that the smaller volume of irrigant in the apical region may have also contributed to the increased final internal temperature with the use of the UX activation system. Furthermore, all systems resulted in a greater rise in irrigant temperature when the initial solution was at 45°C compared to results obtained with the solution at 25°C. Our findings are consistent with those of a previous study [9], where preheating the solution to 60°C led to significantly higher temperatures compared to irrigant at 20°C and 40°C, regardless of the activation method used. The increased NaOCl solution temperature inside the root canal improves its reactivity and disinfect power [11, 12]. However, higher temperatures may reach the outer surface of the roots, affecting the adjacent tissues [7]. Studies [7, 8] have reported that temperatures higher than 47°C may cause severe damage to the periodontal structures. In the present study, the maximum internal temperature was 51.3°C, and the maximum external temperature was 41.7°C. Thus, it may be suggested that dentin acts as a good insulator, and the adjacent tissues may dissipate heating and buffer higher temperatures [1, 18]. In light of this, overall, the external temperature was not affected by the root thirds. Therefore, the use of irrigant preheated to 45°C seems safe.

The data indicate that the external temperature is consistently lower than the intracanal temperature, which reinforces the safety of preheated NaOCl under laboratory conditions. Even with prolonged irrigation (25 s), the heat accumulated in the canal wall was quickly dissipated [18]. Saunders [19] had already indicated that, within the observed temperature range, any potential thermal damage would not compromise the healing of periapical tissues in animal models, as the internally generated heat dissipates rapidly. Furthermore, a previous study [20] demonstrated that the original temperatures of the sodium hypochlorite solutions were quickly attenuated within the root canal, rapidly reaching thermal equilibrium. These findings enhance the understanding of the thermal behavior of irrigant solutions in root canals [20].

The efficacy of NaOCl is directly related to its concentration, temperature, activation period, and renewal [3, 20, 21]. In this study, the activation periods of 20, 30, and 60 s were chosen based on their alignment with current clinical practice in endodontics and other studies published with similar methodology [3, 9, 13]. These intervals are commonly used during irrigation activation to ensure effective debridement and disinfection of the root canal system while maintaining a practical and efficient workflow during treatment [21–23]. It was observed that activation periods (20, 30, and 60 s) little affect the temperature along the root canal. This relative temperature stability may be explained by the period that irrigant remained inside the root canal [15]. Therefore, some authors suggest three cycles of activation for 20 s each to seek constant renewals of the irrigant [22, 23].

The present study has some limitations that warrant discussion. Like other laboratory studies, inherent limitations in the methodological design are present [9, 10]. For instance, the resin used to secure the thermocouples may have introduced artifacts that could have affected temperature readings. However, it was essential for stabilizing and positioning the sensors, ensuring accurate and repeatable measurements. This methodology has been widely validated in previous studies, and it is considered reliable and scientifically sound [11, 12, 18]. Future research should investigate alternative attachment methods to reduce potential artifacts while preserving measurement accuracy. Another limitation is the lack of comparison with other activation systems, which would have provided valuable insights. However, in a previous pilot study, the effect of several activation systems on irrigant temperature was tested. Therefore, the XP Endo-finisher (FKG Dentaire) and the EasyClean insert (Easy) were not employed in the present study, as they did not cause temperature fluctuation during their application inside the root canal [10]. Furthermore, a new device available on the market (UX handpiece) was tested, as it is compact and portable, which facilitates its use. Therefore, it was also aimed to assess whether the use of a system with a higher working frequency was effective and if its performance would be comparable to benchtop ultrasound. Given these experimental limitations, the results of this study should be interpreted with caution, and new methods for analyzing internal and external root temperatures should be developed to properly simulate clinical conditions.

## 5. Conclusion

Considering the conditions of this laboratory study, it was shown that the use of PUA, EA, and UX for the activation of the irrigant, irrespective of the initial temperature evaluated (25°C or 45°C), led to changes in the internal and external radicular temperatures, which may be deemed clinically safe. The activation period had little influence on root temperature changes. EA generated the lowest radicular temperature changes after all activation periods and initial temperatures evaluated.

## Figures and Tables

**Table 1:** Mean values and standard deviation (in parenthesis) of internal radicular temperatures (in °C) obtained by the use of the irrigation activation systems (EA, PUI and UX groups), according to the initial temperature (IT) of the irrigant (25°C and 45°C), period of activation (20-, 30- and 60-s) and root third (cervical, middle, and apical).

Activation period	Activation methods	Initial temperature					
		Cervical		Middle		Apical	
		25°C	45°C	25°C	45°C	25°C	45°C
20 s	EA	32.72 (1.82)^A,a^	36.59 (1.34)^A,b^	33.94 (2.11)^A,a^	37.55 (1.23)^A,b^	34.16 (2.11)^A,a^	37.19 (1.21)^A,b^
	PUI	37.06 (2.16)^B,a^	43.88 (2.99)^B,b^	39.41 (4.56)^B,a^	46.64 (1.94)^B,b^	37.27 (2.58)^AB,a^	44.96 (4.20)^B,b^
	UX	34.54 (2.16)^AB,a^	45.72 (2.56)^B,b^	35.01 (2.27)^Aa^	45.01 (4.18)^B,b^	37.70 (3.09)^B,a^	50.49 (3.62)^C,b^

30 s	EA	33.51 (1.56)^A,a^	36.61 (1.74)^A,b^	34.10 (1.74)^A,a^	**37.66** ^**✔**^ (0.52)^A,b^	34.65 (2.05)^A,a^	37.13 (0.91)^A,a^
	PUI	37.52 (2.24)^B,a^	45.62 (2.16)^B,b^	39.35 (3.21)^B,a^	47.08 (1.97)^B,b^	37.78 (2.66)^AB,a^	45.16 (4.13)^B,b^
	UX	35.98 (1.91)^AB,a^	47.39 (2.29)^B,b^	36.44 (2.42)^AB,a^	46.76 (2.91)^B,b^	38.86 (3.38)^B,a^	**51.35** ^**✔**^ (2.92)^C,b^

60 s	EA	34.31 (1.63)^A,a^	36.38 (2.20)^A,a^	34.78 (1.64)^A,a^	37.42 (0.66)^A^	35.20 (1.44)^A,a^	36.72 (0.89)^A,a^
	PUI	37.07 (2.27)^B,a^	46.89 (2.11)^B,b^	40.54 (3.36)^B,a^	**48.64** ^**✔**^ (2.11)^B^	38.65 (2.82)^AB,a^	46.66 (3.56)^B,b^
	UX	38.19 (3.72)^Ba^	49.49 (3.71)^B,b^	37.97 (2.67)^B,a^	48.60 (2.08)^B^	40.56 (3.72)^B,a^	51.00 (3.74)^C,b^

*Note:* Different superscript uppercase letters indicate a statistically significant difference among the activation methods within the same initial temperature, root third and activation period (*p* < 0.05) (columns). Different superscript lowercase letters indicate a statistically significant difference between the initial temperature within each activation method, root third and activation period (*p* < 0.05) (rows). ^**✔**^ Largest internal temperatures obtained in each activation system group. The values highlighted in bold correspond to the highest temperature value observed in each activation system.

Abbreviations: EA, endoactivator; PUI, passive ultrasonic irrigation; UX, Ultra X.

**Table 2:** Mean values and standard deviation (in parenthesis) of external radicular temperatures (in °C) obtained by the use of the irrigation activation systems (EA, PUI, and UX groups), according to the initial temperature (IT) of the irrigant (25°C and 45°C), period of activation (20-, 30-, and 60 s) and root third (cervical, middle, and apical).

Activation period	Activation methods	Initial temperature					
		Cervical		Middle		Apical	
		25°C	45°C	25°C	45°C	25°C	45°C
20 s	EA	29.77 (1.46) ^A,a^	37.97 (1.15)^A,b^	32.76 (1.24)^A,a^	38.05 (0.64)^A,b^	33.70 (2.47)^A,a^	38.25 (0.99)^A,b^
	PUI	36.95 (2.86) ^B,a^	38.66 (0.40)^A,a^	34.56 (1.62)^A,a^	38.52 (1.84)^A,b^	35.84 (0.82)^AB,a^	38.97 (0.88)^AB,b^
	UX	35.45 (1.90)^B,a^	39.80 (3.29)^A,b^	37.85 (1.96)^B,a^	41.26 (2.39)^B,b^	36.40 (1.44)^B,a^	40.83 (2.22)^B,b^

30 s	EA	29.85 (1.47)^A,a^	38.31 (0.97)^A,b^	33.21 (1.23)^A,a^	37.96 (1.02)^A,b^	33.98 (2.27)^A,a^	38.36 (0.90)^A,b^
	PUI	38.20 (4.13)^B,a^	38.75 (0.52)^A,a^	34.42 (2.08)^A,a^	38.86 (1.39)^A,b^	36.52 (0.92)^B,a^	38.41 (2.36)^A,a^
	UX	36.07 (2.31)^B,a^	40.65 (4.01)^A,b^	38.65 (1.77)^B,a^	41.78 (2.21)^B,b^	37.07 (1.56)^B,a^	41.04 (1.89)^B,b^

60 s	EA	30.28 (0.99)^A,a^	38.03 (1.39)^A,b^	33.59 (1.01)^A,a^	38.20 (1.20)^A,b^	33.70 (1.77)^A,a^	**38.76** ^**✔**^ (0.91)^A,b^
	PUI	39.00 (5.40)^B,a^	39.80 (1.00)^A,a^	36.18 (2.29)^A,a^	39.38 (2.17)^A,b^	37.04 (1.48)^B,a^	**41.26** ^**✔**^ (2.39)^AB,b^
	UX	38.15 (2.13)^B,a^	41.30 (4.21)^A,b^	39.95 (1.71)^B,a^	**42.70** ^**✔**^ (1.68)^B,b^	37.74 (1.65)^B,a^	41.68 (2.67)^B,b^

*Note:* Different superscript uppercase letters indicate a statistically significant difference among the activation methods within the same initial temperature, root third and activation period (*p* < 0.05) (columns). Different superscript lowercase letters indicate a statistically significant difference between the initial temperature within each activation method, root third and activation period (*p* < 0.05) (rows). ^**✔**^ Largest external temperatures obtained in each activation system group. The values highlighted in bold correspond to the highest temperature value observed in each activation system.

Abbreviations: EA, endoactivator; PUI, passive ultrasonic irrigation; UX, Ultra X.

## Data Availability

The data that support the findings of this study are available from the corresponding author upon reasonable request.
